# Effectiveness of a Train-the-Trainer Pedagogical Pilot Workshop in Improving Educators’ Knowledge and Competency in Objective Structured Clinical Examination Implementation

**DOI:** 10.7759/cureus.109894

**Published:** 2026-05-29

**Authors:** Asma Ben Amor, Hassan Farhat, Amina Aounallah, Walid Naija, Aicha Bouaziz, Bassem Charfeddine, Souad Chelbi, Olfa Bouallègue, Mohamed Ben Dhiab

**Affiliations:** 1 Department of Medical Education and Simulation, Higher School of Health Sciences and Techniques, University of Sousse, Sousse, TUN; 2 Laboratory of Epidemiology of Mental Illnesses, Screening and Early Management, Faculty of Medicine "Ibn El Jazzar", University of Sousse, Sousse, TUN; 3 Major Incident Preparedness and Resilience and Ambulance Service Group, Hamad Medical Corporation, Doha, QAT; 4 Department of Dermatology, University Hospital Farhat Hached, Sousse, TUN; 5 Department of Anesthesia and Intensive Care, Faculty of Medicine "Ibn El Jazzar", University of Sousse, Sousse, TUN; 6 Department of Medical Education, Higher School of Health Sciences and Techniques, University of Sousse, Sousse, TUN; 7 Laboratory of Microbiology, Sahloul University Hospital, Sousse, TUN; 8 Department of Forensic Medicine, Faculty of Medicine "Ibn El Jazzar", University of Sousse, Sousse, TUN

**Keywords:** continuing medical education, educator competency, faculty development, knowledge improvement, objective structured clinical examination, pedagogical intervention, train-the-trainer, tunisia

## Abstract

Background: The Objective Structured Clinical Examination (OSCE) was implemented at the School of Health Sciences and Technologies of Sousse, Tunisia, without prior educator training, raising concerns regarding assessment quality and stakeholder satisfaction. This study evaluated the effectiveness of a pedagogical pilot training workshop in improving educators’ knowledge of OSCE principles and their satisfaction with the intervention.

Methodology: This single-group pre- and post-interventional study involved 20 of 30 invited educators (participation rate: 20/30, 66.7%) who completed pre- and post-training knowledge assessments comprising 24 multiple-choice questions covering OSCE domains, competencies, reliability measures, and station design. All 20 participants (20/20, 100%) completed a 24-item satisfaction survey after the training. Descriptive analyses, Shewhart control charts, boxplots, Spearman rank correlations, and multivariate linear regression were employed.

Results: Among the 20 participants, mean knowledge scores increased significantly from 10.27/20 (SD = 3.81) before training to 17.27/20 (SD = 2.12) after training, with pass rates improving from 11/20 (55%) to 20/20 (100%). Perfect post-training scores (20/20, 100%) were achieved in foundational domains (OSCE domains assessed, competencies, and validity recommendations). Satisfaction analysis revealed strong associations between organizational aspects, pedagogical quality, and general appreciation and global satisfaction (r = 0.70-0.94, p < 0.05). Multivariate regression identified three key predictive factors: learning atmosphere (β = 0.25), room comfort (β = 0.21), and trainer approachability (β = 0.38).

Conclusions: The train-the-trainer pilot workshop represents an effective continuing medical education strategy for healthcare professionals. The intervention successfully addressed educators’ knowledge deficits while maintaining high satisfaction across demographic groups, supporting institutional implementation across diverse healthcare education contexts.

## Introduction

Assessing clinical skills is a fundamental issue in the training of healthcare professionals. The Objective Structured Clinical Examination (OSCE) has been identified as one of the most important methods for assessing students in medical education and has become a preferred approach in many educational settings for measuring skills. It offers a standardized, objective approach to assessing students under specific conditions, which reduces subjectivity, ensures a fair evaluation of clinical competencies, and provides accurate feedback on the clinical skills acquired by students [1].

The OSCE was introduced in the School of Health Sciences and Technologies of Sousse in Tunisia in 2019 under difficult financial circumstances and amid COVID-19 pandemic restrictions, during which in-hospital clinical examinations were considerably limited. This led to a rapid implementation of the OSCE without prior training for educators or adequate briefing for students. This abrupt transition raised concerns regarding assessment quality and students’ satisfaction with the new evaluation method.

Therefore, it was essential to assess educators’ and students’ satisfaction with the OSCE to identify gaps as perceived by stakeholders, as reported in previously published research [2]. Nevertheless, various challenges were identified that limited educators’ and students’ satisfaction with the OSCE, including variation in satisfaction attributable to difficulties in conducting certain OSCE stations properly [2].

In the literature, challenges in healthcare professionals’ continuous training have long been recognized. After graduation, practicing healthcare professionals in many countries pursue continuing medical education (CME) to maintain their skills at optimal levels, keep abreast of new technologies, and meet license renewal requirements [3]. Traditional examples of CME have included conferences, workshops, and large lectures. CME requirements are not always available in low-income countries such as Tunisia, making it necessary to empower the existing educational methods. Furthermore, no single educational method has been identified as producing the best outcome on its own [4,5].

Providing adequate OSCE-focused training for health sciences educators in the Higher School of Health Sciences and Techniques of Sousse was therefore essential to mitigate the challenges encountered during earlier OSCE implementation [2]. A "train-the-trainer" pilot workshop was designed to help fill previously identified gaps, optimize the OSCE evaluation process, and promote a culture of educational excellence among the School’s educators.

Accordingly, this study aimed to evaluate the effectiveness of the train-the-trainer workshop in improving educators’ OSCE knowledge. It also aimed to assess educators’ satisfaction with the pilot train-the-trainer training session provided, identify which subdimensions in the satisfaction questionnaire and item-level factors most strongly drive global satisfaction, examine whether demographic and professional characteristics influenced satisfaction, and evaluate the acceptability/feasibility of the approach as a scalable faculty-development strategy in resource-limited settings.

## Materials and methods

This was an interventional, cross-sectional study conducted at the School of Health Sciences and Technologies of Sousse, Tunisia. It assessed educators’ knowledge before and after a pedagogical training workshop designed to improve educator competencies in organizing OSCE sessions. The pedagogical pilot training session was delivered by two recognized experts in simulation-based medical education from the Faculty of Medicine "Ibn El-Jazzar" of Sousse, Tunisia (Prof. W.N. and Prof. A.A.), who are co-authors of this article.

The pedagogical training session was organized on May 24, 2024, over four hours according to the agenda detailed in Table 1. The session included five interactive activities: creating a specification table, writing an OSCE station, developing an observation grid, designing a simulated patient scenario, and discussing the organizational aspects of the OSCE. Each activity lasted 50 minutes and included a phase of individual work, group work, and a plenary session to share results. A break was scheduled halfway through to encourage informal exchanges between participants. This structured, collaborative program enabled educators to develop practical skills while consolidating their theoretical knowledge about the OSCE.

**Table 1:** Pedagogical training session organization OSCE: Objective Structured Clinical Examination

Time	Activity	Details
09:00	Welcome	-
09:20	Introduction: the role of OSCE in learning assessment	Presentation
09:30	Activity 1: Specification table	Individual work: 10 min; group work: 15 min; plenary session: 25 min
10:20	Activity 2: Writing an OSCE station	Individual work: 10 min; group work: 15 min; plenary session: 25 min
11:10–11:20	Coffee break	-
11:20	Activity 3: Observation checklist	Individual work: 10 min; group work: 15 min; plenary session: 25 min
12:10	Activity 4: Simulated patient (SP) scenario	Presentation: 10 min; group work: 25 min; plenary session: 25 min
13:00	Activity 5: Organizational aspects of an OSCE examination	Presentation: 10 min; group work: 25 min; plenary session: 25 min

Testing the change idea

The implementation of the pedagogical pilot training session followed the Plan-Do-Study-Act (PDSA) cycle developed by W. Edwards Deming, which provided a structured framework for the effective implementation and evaluation of this educational intervention.

Plan

The objective was to improve educators’ knowledge of OSCE principles and enhance their satisfaction with clinical assessment training. Key components included the design of a one-day training seminar with five interactive activities focused on OSCE station creation, evaluation grids, and standardized patient scenarios; the development of two validated surveys (through content validation using Aiken’s V coefficient for content validaton), a 24-item multiple-choice knowledge assessment covering OSCE domains, skills, reliability measures, and station design; a 24-item Likert-scale satisfaction survey evaluating organization, content relevance, and practical impact); and the engagement of 20 educators (N = 20) from emergency and critical care (8/20, 40%) and other specialties (12/20, 60%) with varied OSCE experience.

Do

The agenda of the pedagogical session is described in Table 1. A pre-training knowledge assessment was administered one week prior to the workshop. Expert-led sessions included structured individual and group work phases. The post-training knowledge test and satisfaction survey were completed immediately after the session.

Study

Success metrics were established using a pre- and post-training knowledge questionnaire with 24 multiple-choice questions covering OSCE concepts, along with a satisfaction questionnaire evaluating organizational aspects, content relevance, pedagogical quality, and practical impact using a Likert scale.

Act

The improvement strategy was to decide whether to sustain or further improve the implemented training based on participants’ knowledge gains and satisfaction.

Survey validation

An assessment was administered to educators before the training session to establish a baseline of their knowledge of OSCE principles and practices. An identical assessment was distributed after the training to measure changes in understanding. A satisfaction questionnaire was also administered at the end of the session. Two experts validated the satisfaction survey. Validation results were evaluated using the Aiken V coefficient for content validation and percent agreement for inter-rater agreement, assessing whether each item was pertinent to the study objective, a good indicator of the construct of interest, and clear to the respondent.

The 24-item multiple-choice knowledge assessment was developed by the research team based on the OSCE literature and locally identified knowledge gaps from prior work. The multiple-choice knowledge assessment was face-validated by two independent experts in simulation-based medical education. The knowledge assessment covered various aspects of OSCE knowledge, while the satisfaction survey focused on training-related elements, including content relevance, clarity of presentations, and interaction with trainers. The pre- and post-training knowledge assessment comprised 24 multiple-choice questions. Each item carried equal weight, with a total possible score of 20 points (each correct answer scoring 0.83 points, summed and reported on a 20-point scale), and addressed critical themes integral to the effective implementation of OSCE. These themes included identifying cognitive, psychoaffective, and psychomotor domains assessed by OSCE; the specific skills evaluated (such as history-taking, interpretation of complementary examinations, communication, and care organization); and measures to enhance the reliability of OSCE assessments. Additional items explored participants’ comprehension of the role and types of standardized patients, distinctions between formative and summative uses of OSCE stations, and recommendations for ensuring validity in station design. Other items covered essential criteria for scoring grids, station sequences, and construction stages, alongside an analysis of the terms "structured" and "objective" within the OSCE framework.

Population and sampling

A convenient sampling strategy was utilized. The target population included 30 educators involved in the implementation of OSCE examinations at the school. A total of 20 of 30 invited educators from several disciplines attended the session, representing a participation rate of 20/30 (66.7%). All 20 attendees completed both the pre- and post-training assessments and the satisfaction survey (20/20, 100%). Only medical simulation educators at the Higher School of Health Sciences and Techniques of Sousse were considered, ensuring a homogeneous population with shared institutional context and comparable baseline exposure to OSCE practice. The sample of 20 was deemed appropriate for a pilot intervention to assess feasibility, acceptability, and preliminary effectiveness prior to broader implementation.

Data analysis

Descriptive analyses were conducted on the pre- and post-training knowledge assessments to evaluate the intervention’s impact on educators’ knowledge gains. Descriptive analyses of the satisfaction questionnaire assessed participants’ satisfaction with the pedagogical workshop.

For the pre- and post-workshop knowledge assessments, Shewhart control charts were used to evaluate variation in educators’ performance before and after the training intervention [6]. Compared with traditional hypothesis testing, the Shewhart control chart approach provides a visual representation of process stability and of meaningful changes over time, enabling practical detection of trends that conventional statistical tests might overlook. Each control chart displayed a central line representing the mean score, alongside upper control limits (UCL) and lower control limits (LCL) positioned at three sigma (σ) from the mean, thereby defining the expected bounds of normal variation (common-cause variation) [6,7]. Data points falling outside these limits signaled special-cause variation requiring investigation, while points within the limits reflected inherent system variability [7]. This approach emphasizes practical relevance and empirical process improvement rather than statistical significance alone.

To complement the control chart analysis, boxplots were constructed to visualize the distribution of evaluation scores across the study phases. These graphical displays presented five key summary statistics: minimum value, first quartile (Q1), median, third quartile (Q3), and maximum value. The interquartile range (IQR), represented by the box itself, quantified score dispersion and enabled comparison of score distributions between pre- and post-intervention periods.

For the educators’ satisfaction questionnaire, Spearman rank correlation tests and linear regression analyses were applied, enabling comparable interpretation across both study components. Results from Spearman correlations and linear regression analyses were presented using heatmaps to illustrate correlations and p-values among satisfaction scores, thematic domains, and demographic variables. Bar charts complemented these heatmaps by displaying regression coefficients, p-values, and R-squared values for each satisfaction theme to provide a comparative analysis of associations, correlations, and distributions across groups, relative to traditional tabular formats that often present excessive information with limited interpretive utility.

Ethical considerations

Participation in the pilot workshop and surveys was voluntary. Educators provided informed consent before completing the pre-training assessment. All data were anonymized before analysis. The study protocol was reviewed and approved by the institutional review committee of the Faculty of Medicine "Ibn El Jazzar," University of Sousse, Tunisia.

## Results

Satisfaction survey validation

Table 2 presents the satisfaction survey validation results. All items achieved an Aiken V coefficient of 0.90 or higher, indicating excellent validation across clarity, pertinence, and quality as indicators. An Aiken V ≥ 0.80 indicates excellent content validation. Percent agreement ranged from 47.6% (clarity) to 71.4% (pertinence).

**Table 2:** Satisfaction survey validation results

Characteristic	Percent agreement (%)	Agreement level	Aiken V (validation level)
Clarity	47.60	Moderate	0.90 (excellent)
Pertinence	71.40	Good	0.94 (excellent)
Good indicator	66.70	Good	0.94 (excellent)

Participant characteristics

As shown in Table 3, of the 20 participants, the study population was predominantly female (16/20, 80%) and included four men (4/20, 20%). Nearly half of the participants (9/20, 45%) were aged 37-43 years, and 9/20 (45%) had fewer than six years of professional seniority. Eight of the 20 educators (8/20, 40%) belonged to the emergency and resuscitation section. The most common grade was technician major (5/20, 25%). Fifteen of the 20 participants (15/20, 75%) held a research master’s degree in health sciences. Thirteen respondents (13/20, 65%) reported having previously taken part in an OSCE; among these 13, 4/13 (30.77%) had taken part only once.

**Table 3:** Distribution of educators by sociodemographic and professional characteristics (N = 20) OSCE: Objective Structured Clinical Examination

Characteristic	n	%
Gender		
Men	4	20
Women	16	80
Age (years)		
23-29	7	35
30-36	2	10
37-43	9	45
44-50	1	5
>50	1	5
Seniority (years)		
<6	9	45
6-12	4	20
13-19	7	35
Section		
Anesthesia	2	10
Pediatrics	4	20
Health sciences	2	10
Midwifery	1	5
Emergency and resuscitation	8	40
Nursing	3	15
Grade		
Educator	4	20
Nurse major	1	5
Research master	1	5
PhD	3	15
Principal health educator	4	20
Major technician	5	25
Senior technician	2	10
Highest degree obtained		
PhD	1	5
Applied license	2	10
Research master	15	75
Principal health educator degree	2	10
Prior OSCE participation		
Yes	13	65
No	7	35
Number of OSCE sessions participated (among those with prior experience)		
1 time	4	30.77
2 times	3	23.08
3 times	1	7.69
4 times	3	23.08
5 times	1	7.69
6 times	1	7.69

Knowledge improvement

**Figure 1:**
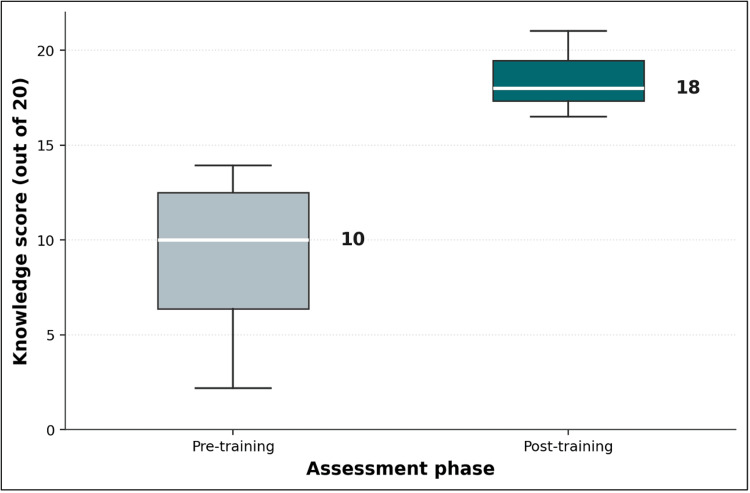
Boxplots of pre- and post-training knowledge assessment scores (N = 20)

**Figure 2:**
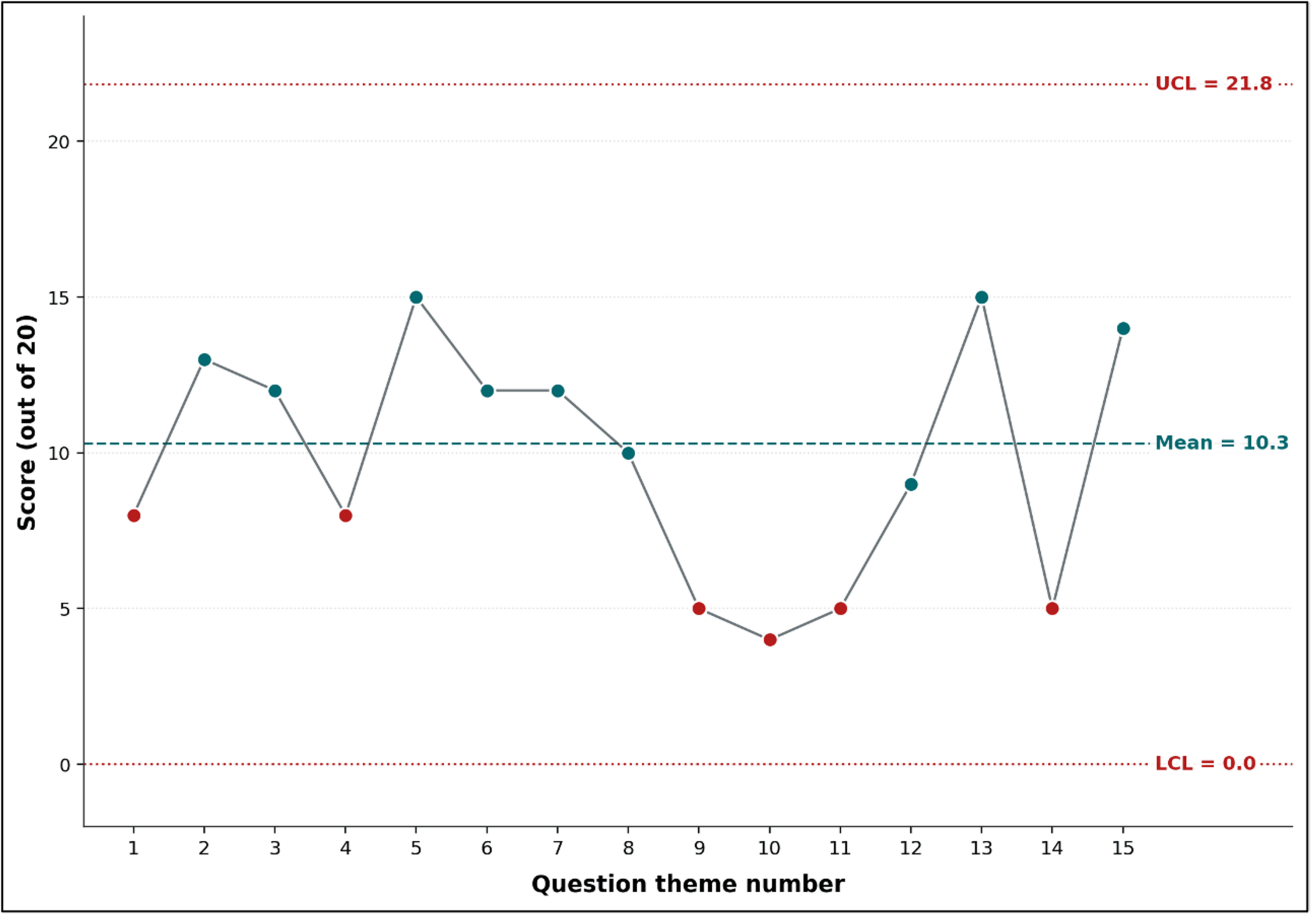
Shewhart control chart of pre-training knowledge scores (N = 20) across the 15 question themes

**Figure 3:**
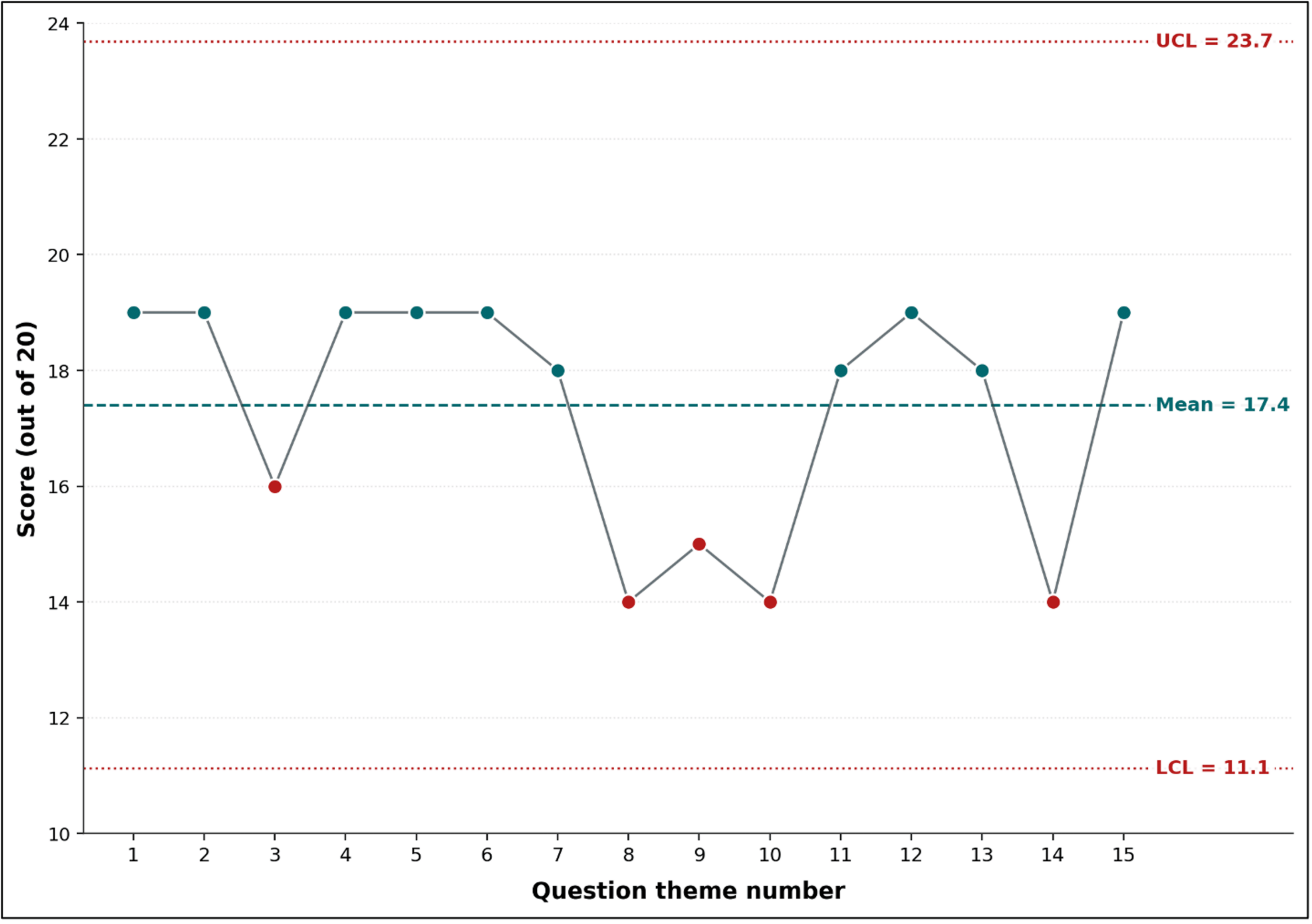
Shewhart control chart of post-training knowledge scores (N = 20) across the 15 question themes

Figures 1-3 illustrate the progression of educators’ knowledge and understanding of OSCE principles before and after the pedagogical training session. Figure 1 presents boxplots of the pre- and post-training knowledge assessments, highlighting a substantial score improvement. The median score increased from 10/20 to 18/20, with reduced variability in post-training results, indicating a more consistent level of understanding among participants. Figure 2, a Shewhart chart of pre-training scores, reveals marked variation across different question themes, with several items falling below the mean score (10.3). Notably, concepts such as the meaning of "structured" and "objective" and the characteristics of scoring grids scored particularly low, reflecting gaps in foundational knowledge before training. In contrast, Figure 3, which depicts post-training scores using the same Shewhart chart format, demonstrates substantial improvements across all themes. Most items exceeded the mean score (17.4), with minimal variation and no items falling below the lower control limit. This suggests that the training effectively addressed initial knowledge deficits while enhancing participants’ comprehension of critical OSCE concepts. The overall consistency and upward shift in scores across Figures 1-3 underscore the success of the training intervention in achieving its educational objectives.

In Figure 1, the horizontal line within each box marks the median; box edges represent the first and third quartiles; whiskers extend to the minimum and maximum values. In Figure 2, the dashed line represents the mean score (10.3/20); dotted lines mark upper and lower control limits at ±3 sigma. Question themes (x-axis): 1, domains assessed by OSCE; 2, skills assessed by OSCE; 3, measures to increase OSCE reliability; 4, standardized-patient types; 5, use of OSCE stations; 6, recommendations for improved OSCE validity; 7, evaluation criteria in an OSCE exam; 8, concept of "station" in an OSCE exam; 9, meaning of the word "structured"; 10, meaning of the word "objective"; 11, number of elements in the correction grid; 12, nature of criteria in the correction grid; 13, characteristics of the correction grid; 14, person who can ask questions at an OSCE station; and 15, order of steps in constructing an OSCE station.

In Figure 3, the dashed line represents the mean score (17.4/20); dotted lines mark upper and lower control limits at ±3 sigma. Question themes (x-axis) included the following: 1, domains assessed by OSCE; 2, skills assessed by OSCE; 3, measures to increase OSCE reliability; 4, standardized-patient types; 5, use of OSCE stations; 6, recommendations for improved OSCE validity; 7, evaluation criteria in an OSCE exam; 8, concept of "station" in an OSCE exam; 9, meaning of the word "structured"; 10, meaning of the word "objective"; 11, number of elements in the correction grid; 12, nature of criteria in the correction grid; 13, characteristics of the correction grid; 14, person who can ask questions at an OSCE station; and 15, order of steps in constructing an OSCE station.

Detailed item-level results are reported in Appendix 1 (denominator: N = 20 participants). Before the training, participants demonstrated limited awareness of several key OSCE concepts: 18/20 (90%) correctly identified that OSCE assesses cognitive, psychoaffective, and psychomotor domains simultaneously; this figure rose to 20/20 (100%) after training, indicating full mastery of this concept. Recognition of assessable skills (such as history-taking, interpretation of complementary examinations, and communication) improved substantially, with all 20 participants (20/20, 100%) correctly identifying these skills after training. Understanding of standardized patient roles also progressed markedly, with recognition of standardized-patient types, including real patients, trained individuals, and paid actors, rising from 8/20 (40%) to 18/20 (90%). Participants showed enhanced comprehension of OSCE station applications, with 16/20 (80%) correctly acknowledging their use for both formative and summative assessments after training. Recommendations for improving OSCE validity were better understood after the intervention, with all 20 participants (20/20, 100%) correctly recognizing the importance of designing short-duration stations; however, misconceptions about station duration persisted among 13/20 participants (65%). Evaluation criteria also showed notable progress, with recognition that criteria must adhere to the all-or-nothing principle increasing from 13/20 (65%) to 20/20 (100%). Understanding of the "structured" and "objective" concepts improved significantly, although some confusion remained regarding their precise definitions.

Overall, across the 20 participants, the training resulted in a substantial increase in knowledge scores, from a mean of 10.27/20 (SD = 3.81) before training to 17.27/20 (SD = 2.12) after training, and a pass-rate improvement from 11/20 (55%) to 20/20 (100%), demonstrating its effectiveness in addressing educators’ knowledge gaps and enhancing their confidence in applying OSCE methodology.

Satisfaction with the pedagogical workshop

**Figure 4:**
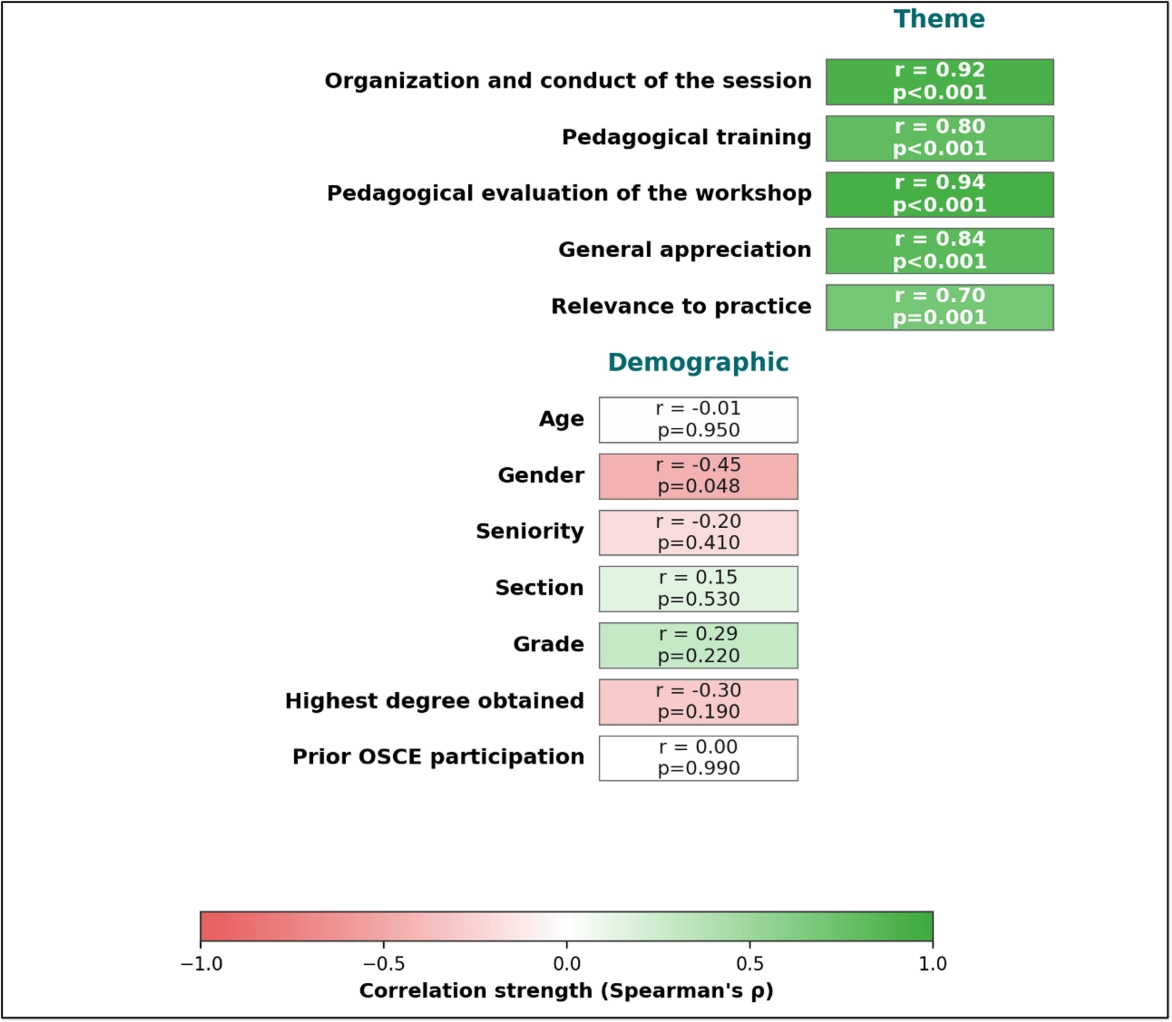
Associations between global satisfaction score, thematic domains, and demographic variables (Spearman’s ρ with corresponding p-values; N = 20)

The mean global satisfaction score was 4.37/5. Figure 4 presents the bivariate correlation analysis, which revealed significant associations between satisfaction dimensions and global satisfaction ratings. Organizational aspects, pedagogical training quality, pedagogical evaluation of the workshop, general appreciation, and relevance to practice demonstrated strong positive correlations with global satisfaction, with coefficients ranging from r = 0.70 to r = 0.94 (p < 0.05). Demographic variables, including age, seniority, academic section, grade, highest degree obtained, and prior OSCE participation, were not significantly correlated with satisfaction (p > 0.05). Gender demonstrated a statistically significant negative correlation with satisfaction (r = -0.45, p < 0.05), with female participants reporting higher mean satisfaction scores than male participants. Cell color encodes correlation strength as shown in the color bar.

**Figure 5:**
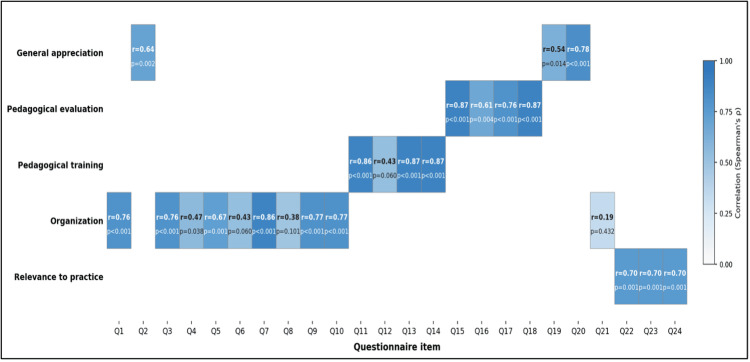
Item-level bivariate correlations with global satisfaction (Spearman’s ρ with p-values; N = 20)

Figure 5 presents the item-level correlation analysis, which revealed differential patterns of association with global satisfaction. Items within the domains of organizational aspects, pedagogical evaluation, and general appreciation exhibited the strongest correlations (r = 0.87, p < 0.05), whereas items outside these thematic domains demonstrated weaker correlations and did not consistently reach statistical significance (p > 0.05). Items Q1-Q24 from the satisfaction questionnaire are shown along the x-axis.

**Figure 6:**
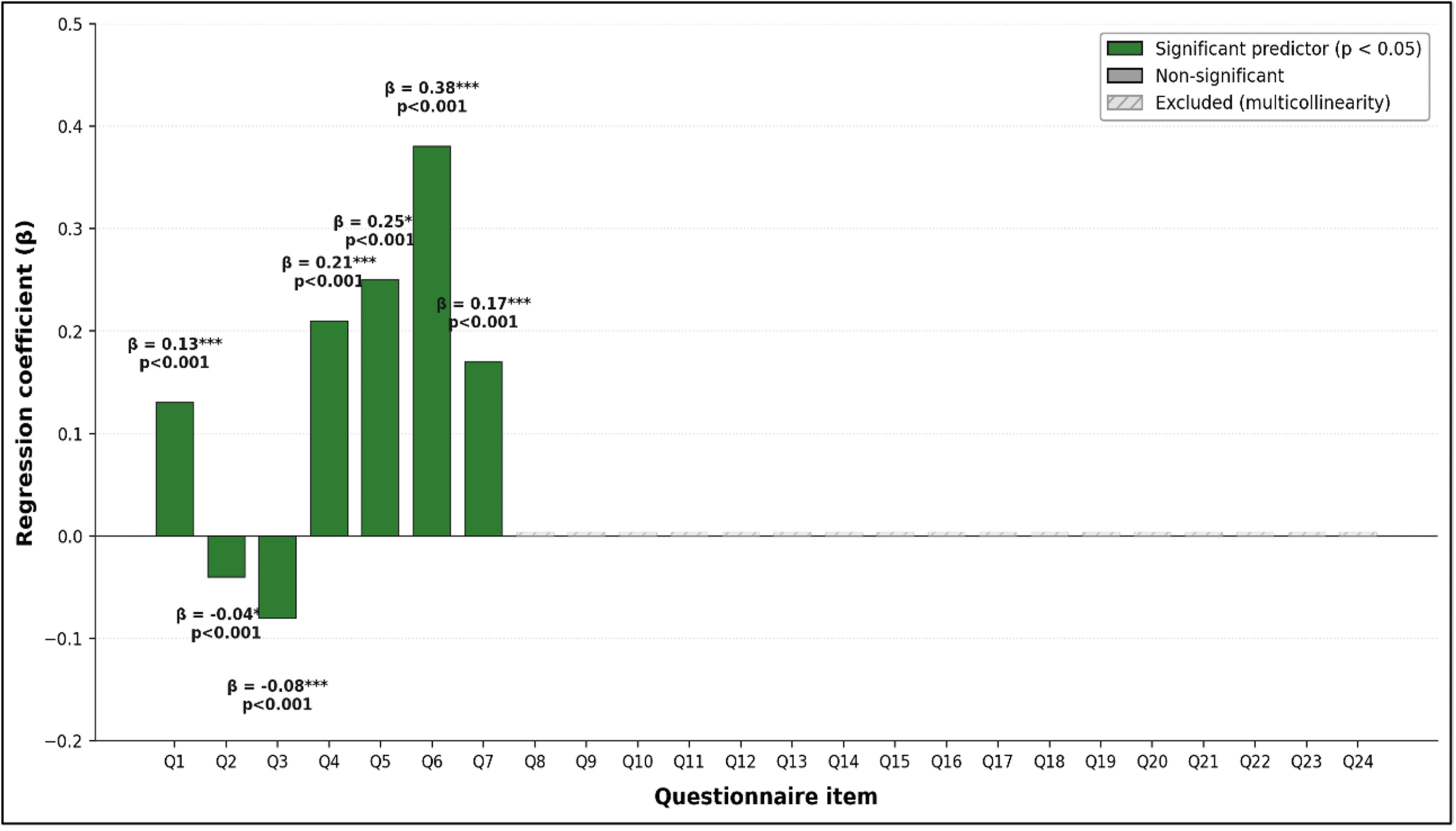
Multivariate regression analysis: item-level predictors of global satisfaction (N = 20)

Figure 6 presents the multivariate regression analysis, which identified three items with significant independent predictive contributions to global satisfaction scores. Item Q5 (general atmosphere conducive to learning) yielded β = 0.25 (p < 0.05), Item Q6 (training room appropriateness and comfort) yielded β = 0.21 (p < 0.05), and Item Q7 (trainer welcoming attitude) yielded β = 0.38 (p < 0.05). Item Q3 (trainer availability) demonstrated a significant negative coefficient (β < 0, p < 0.05), indicating an inverse relationship with global satisfaction in the multivariate model. Multiple remaining items failed to demonstrate significant independent predictive coefficients (p > 0.05), suggesting that their bivariate associations with global satisfaction were mediated through, or redundant with, variables retained in the final model.

Significance coding is as follows: *** p < 0.001, ** p < 0.01, * p < 0.05. Items Q8-Q24 were excluded from the final multivariate model due to multicollinearity with retained predictors; their bivariate associations with global satisfaction (Figure 5) were mediated through the variables retained above.

## Discussion

The School of Health Sciences and Technologies of Sousse introduced the OSCE as a new pedagogical assessment method, mainly under the constraints of the COVID-19 pandemic, which made it challenging to access hospitals and apply traditional bedside clinical examinations. The success of this approach depends largely on its correct implementation by educators and on their thorough understanding of the underlying pedagogical principles. To meet these needs, an educators’ training course was organized to provide essential foundational training in medical education.

Train-the-trainer workshops are widely recognized as an effective tool for continuing medical education among healthcare professionals across various fields of disease education and management [8,9]. They offer educators the opportunity to clarify doubts and strengthen their understanding. In this context, train-the-trainer workshops provide a coherent framework, educational tools, and collective motivation to learn, making this a feasible and effective strategy for disseminating and integrating medical education practices.

The OSCE is recognized as an essential assessment tool in medical education, enabling the evaluation not only of students’ clinical skills but also of their ability to interact with standardized patients in a simulated environment [10,11]. Our results show that educators significantly improved their understanding of the areas assessed by the OSCE, the required skills, and the measures needed to enhance reliability. For example, the correct identification of the domains assessed by the OSCE rose from 8/20 participants (40%) before training to 20/20 (100%) afterward, indicating complete mastery of the subject.

The results concerning educators’ knowledge of the OSCE before and after training reveal a significant improvement in their understanding of key concepts associated with this assessment method. Before training, educators’ mean score was 10.27/20, rising to 17.27/20 after training, a substantial gain in knowledge. This change is corroborated by the pass rate among the 20 participants, which rose from 11/20 (55%) to 20/20 (100%), underlining the effectiveness of the training.

However, some items, such as "measures that increase the reliability of an OSCE," showed a less marked improvement, with the number of correct responses rising from 13/20 participants (65%) to 16/20 (80%). This suggests that, despite the general improvement, certain technical or theoretical aspects still require particular attention in future training courses. In addition, although the understanding of the "station" concept improved from 9/20 (45%) to 14/20 (70%), there is still room to improve to ensure that all educators have a uniform understanding of this key term in the context of OSCE.

The use of a formative approach in OSCE stations not only enables the assessment of students’ skills but also provides immediate feedback, thereby promoting continuous learning [12]. These findings underscore the importance of integrating such assessment methods into the learning curriculum to prepare students effectively for clinical realities. Previous work has also linked job satisfaction more broadly to the gap between aspirations and what is actually encountered in practice [13], suggesting that aligning expectations with the practical and theoretical learning offered is important when designing training of this kind. Recent evidence supports the sustained impact of train-the-trainer interventions on professional development among nurses and allied health educators [14], and a systematic review with meta-analysis has shown that structured train-the-trainer interventions yield measurable gains in trainers’ learning and teaching behaviors when educating health care professionals [15].

The OSCE pedagogical workshop received an overall positive evaluation, with a mean satisfaction score of 4.37/5, reflecting participants’ favorable perceptions of its organization, pedagogical content, and practical relevance. While the majority appreciated the duration and availability of trainers, only 13/20 (65%) felt that time distribution between activities was optimal, highlighting an area for improvement. Pedagogical content was deemed relevant by most participants; however, only 14/20 (70%) felt they had effectively consolidated their OSCE assessment prerequisites, suggesting the need for enhanced focus on practical demonstrations and real-world simulations to bridge theoretical concepts with application. Interactive activities such as group work were well-received, with 16/20 (80%) agreeing that they fostered collaboration and dynamic learning. The overall quality of the pedagogical workshop was rated highly (19/20, 95%), and participants expressed confidence in its potential to improve their professional practice.

With regard to session organization (Item 1), the majority of participants (15/20, 75%) felt that the duration was well suited to the content presented, and a similar proportion considered the trainers to be available. However, only 13/20 (65%) felt that the time between activities was well distributed, suggesting that further optimization of this organizational dimension is needed. Regarding pedagogical training, although the theme was deemed relevant by most participants in terms of personal or professional development, only 14/20 (70%) felt they had effectively consolidated their OSCE assessment prerequisites. This result highlights the need to strengthen the specific pedagogical content linked to the OSCE to better meet participants’ expectations. During the workshop, participants had the opportunity to reinforce their knowledge of OSCE station design and planning, notably through group work that encouraged collaboration and exchange of ideas. These interactive activities enabled dynamic learning, enriching participants’ understanding and mastery of the skills required for effective evaluation [16].

These findings align with evidence from previous studies demonstrating that OSCE-focused training workshops significantly enhance educators’ confidence and skills [16]. To further optimize learning, it would be appropriate to integrate practical demonstrations into future training courses, using videos of OSCE stations or live OSCE simulations to enable participants to observe and practice the concepts covered; recent evidence also supports structured training of raters and examinees, notably through simulation-based approaches, as the most effective way to improve OSCE conduct [17]. Synchronous workshop-based feedback models have similarly been shown to support faculty development in OSCE station design [18]. When identified gaps are addressed and innovative tools are integrated, future workshops can further optimize educators’ readiness to implement the OSCE effectively in diverse educational contexts [18]. The pedagogical evaluation of the seminar showed strong agreement regarding the interactivity of the sessions and the relevance of the documents provided; approximately 16/20 participants (80%) appreciated these specific aspects, reflecting high quality in these dimensions.

Beyond the immediate training context, the value of structured faculty development is particularly salient in resource-limited settings, where systematic investment in educator capacity has been identified as a key lever for strengthening medical education systems [19]. A recent systematic review of undergraduate nursing education further confirms that OSCE- and standardized-patient-based experiences consistently enhance clinical competence, confidence, and learner satisfaction across programs, reinforcing the rationale for investing in educator preparedness [20].

Limitations

This study has several limitations. The sample consisted of 20 educators from a single institution, limiting generalizability to broader healthcare education contexts. In addition, the nonreliance on real patients reflected practical and cultural constraints inherent to low- and middle-income settings, where recruiting real patients for training is often limited by cultural reluctance to participate in educational scenarios and by the absence of financial compensation mechanisms. The single-group pre-post design without a control group precludes assessment of knowledge retention beyond the immediate post-intervention period. Satisfaction data relied on self-reported measures without external validation. The heatmap visualization format, while intuitive, may not capture more complex satisfaction relationships. The absence of a control group prevents exclusive attribution of improvements to the intervention versus confounding factors. Additionally, qualitative data exploring participants’ learning experiences and barriers were not collected, limiting understanding of underlying mechanisms. Future research should employ larger, multi-institutional samples with longitudinal follow-up and mixed-methods designs.

## Conclusions

The implementation of a train-the-trainer pilot workshop to improve knowledge and skills in conducting OSCE proved to be a feasible and effective strategy. An interactive, hands-on approach rich in exercises is recommended, and our study provides a practical blueprint for such initiatives. To further optimize these workshops, it would be helpful to adjust the time allocation between activities, reinforce content linked to OSCE prerequisites, and foster a welcoming environment to enhance the atmosphere conducive to learning. A post-session follow-up would enable evaluation of the application of acquired skills in daily practice. By integrating these adjustments and reinforcing current strengths, such as interactivity, greater participant satisfaction, and a lasting professional impact can be achieved. A follow-up workshop could also be organized to explore the obstacles encountered and gather participant feedback on their implementation.

## Appendices

Appendix 1 

**Table 4:** Detailed descriptive results of the pre- and post-training knowledge assessments

Question	Items	Réponse
1) Parmi les domaines suivants, lesquels pourraient-ils être évalués par l’ECOS?	Uniquement le domaine cognitif	
	Uniquement le domaine psycho-affectif	
	Uniquement le domaine psychomoteur	
	les trois domaine à la fois	
2) Selon vous, lesquelles des compétences suivantes pourraient être évaluées par l’ECOS?	Anamnèse	
	Interprétation d’examens complémentaires	
	Communication	
	Organisation de soin	
3) Selon vous, laquelle des mesures suivantes augmente la *fiabilité d’un ECOS?	réduire le nombre de stations	
	Standardisation des patients	
	Normalisation (standardisation) des évaluations	
	Réduire l’anxiété des étudiants	
4) Selon vous, le patient standardisé peut être	un vrai patient invité à participer aux ECOS	
	une personne sans expérience particulière et qui sera formé par l’équipe pédagogique	
	- payé pour assurer ce rôle	
	- une personne qui a bénéficié d’une formation académique en santé (étudiant en santé, soignant...)	
5) Les stations ECOS peuvent être utilisés de façon	- seulement formative	
	seulement summative	
	Formative et summative	
6) Pour une meilleure validité d’un ECOS il est recommandé de	Concevoir des stations de courte durée: ( 5 minutes)	
	Minimiser le nombre des stations	
	Augmenter la durée de stations pour contenir un maximum des tâches	
7) Dans un examen ECOS	Le temps déterminé pour réaliser la tâche doit être réaliste	
	La réalisation de la tâche proposée doit faire appel à un champ des connaissances très restreint et spécifique	
	Les critères d’évaluation doivent permettre une certaine liberté de jugement pour l’évaluateur	
	Les critères d’évaluation doivent répondre à la loi du tout ou rien (fait /non fait)	
8)Dans un ECOS	la station consiste à évaluer l’atteinte d’un objectif psychomoteur	
	la station peut se limiter à répondre à évaluer l’atteinte d’un objectif cognitif	
	Une station pause peut intercaler deux stations examen	
9) Dans la nomination « examen clinique objectif structuré » les mots « structuré » signifie	chaque station de l’examen a une tâche très spécifique	
	Chaque station répond à une structure prédéterminée	
	L’examen couvre des compétences clinique structurés	
10) Dans la nomination « examen clinique objectif structuré » les mots « Objectif » signifie	atténuation des facteurs dus à la subjectivité (observation/correction)	
	contenu normalize	
	Une grille d’évaluation est obligatoire pour chaque station	
	Construit selon des objectifs d’apprentissage	
11) En règle générale, pour les stations de courte durée (cinq à sept minutes), dans la grille de correction on peut compter	03 à 10 éléments	
	08 à 25 éléments	
	Entre 10 et 20 éléments	
12) Chaque composante (critère) de la grille d’évaluation:	Doit englober une tâche complexe	
	Doit être précis et concis	
	Peut contenir plus qu’un seul élément à évaluer	
	ne devrait porter que sur un seul élément à évaluer	
13)Une grille de correction:	Peut ne pas être exhaustive	
	Doit être exhaustive	
14)Dans toute station:	L’examinateur peut poser une ou plusieurs questions orales	
	le Patient standardisé peut poser des questions à l’étudiant pendant la rencontre selon le scénario préétabli	
	le candidat peut poser des questions à l’examinateur	
15)Mettre dans l’ordre Les étapes de construction d’une station ECOS Elaborer la grille d’évaluation Rédiger le scénario destiné au patient standardisé(s’il y’en a un) Définir les objectifs Ecrire les directives au candidat Etablir les directives pour les examinateurs Tester la station et la réviser si nécessaire		

Appendix 2 

**Table 5:** Satisfaction questionnaire (full-item list)

Items	1 Pas du tout d’accord	2 Plutôt Pas d’accord	3 neutre	4 Plutôt d’accord	5 Tout à fait d’accord
Organisation et déroulement de la séance					
La durée de l’atelier est adaptée au contenu					
La répartition du temps entre les différentes activités de l’atelier de formation était pertinente					
Les formateurs étaient disponibles					
Le nombre de groupe était convenable					
L’ambiance générale était propice à l’apprentissage					
La salle de formation était appropriée et confortable à la formation					
L’accueil des participants par les formateurs était bienveillant					
Les explications ont été suffisamment claires					
Le temps alloué aux questions était suffisant					
La durée de ce séminaire est suffisante pour une formation adéquate					
Formation pédagogique					
Le thème de la formation est pertinent					
La formation m’a aidé à consolider mes prérequis concernant l’évaluation par ECOS					
Connaissances et compétences des animateurs pertinentes					
Les activités choisies étant en adéquation avec les objectifs pédagogiques					
Evaluation pédagogique de l’atelier					
La séance était interactive					
Les documents fournis ou conseillés sont pertinents					
Les outils pédagogiques étaient bien adaptés					
Travail de groupe et discussion utiles sont favorisés					
Appréciation générale					
La qualité globale de l’atelier est satisfaisante					
Les objectifs définis ont été atteints					
vous recommandez ce séminaire à un collègue					
Pertinence pour la pratique					
La formation m’a permis de mettre à jour mes connaissances					
Cette formation m’a permis d’acquérir des compétences organisationnelles pour planifier une évaluation par ECOS					
La séance peut améliorer ma pratique professionnelle future					

Appendix 3 

**Table 6:** Descriptive results of the satisfaction questionnaire

Items	1 Pas du tout d'accord			2 Plutôt Pas d'accord					3 Neutre				4 Plutôt d'accord							5 Tout à fait d'accord				
Statistiques	Effectif		%	Effectif			%		Effectif			%	Effectif				%			Effectif		%		
Item 1: Organisation et déroulement de la séance																								
1. La durée de l’atelier est adaptée au contenu															5			25		15			75	
2. La répartition du temps entre les différentes activités de l’atelier de formation était pertinente															7			35		13			65	
3. Les formateurs étaient disponibles															5			25		15			75	
4. Le nombre de groupe était convenable															7			35		13			65	
5. L'ambiance générale était propice à l'apprentissage									3			15			4			20		13			65	
6. La salle de formation était appropriée et confortable à la formation															5			25		15			75	
7. L’accueil des participants par les formateurs était bienveillant									2			10			2			10		16			80	
8. Les explications ont été suffisamment claires															4			20		16			80	
9. Le temps alloué aux questions était suffisant															10			50		10			50	
10. La durée de ce séminaire est suffisante pour une formation adéquate															7			35		13			65	
Score Item 1 = 4,36/5																								
Item 2: Formation pédagogique																								
11. Le thème de la formation est pertinent														3		15			17		85			
12. La formation m’a aidé à consolider mes prérequis concernant l’évaluation par ECOS										2	10			4		20			14		70			
13. Connaissances et compétences des animateurs pertinentes														5		25			15		75			
14. Les activités choisies étant en adéquation avec les objectifs pédagogiques										2	10			4		20			14		70			
Score Item 2 = 4,2/5																								
Item 3: Évaluation pédagogique de l’atelier																								
15. La séance était interactive										2	10			2		10			16		80			
16. Les documents fournis ou conseillés sont pertinents														4		20			16		80			
17. Les outils pédagogiques étaient bien adaptés														5		25			15		75			
18. Travail de groupe et discussion utiles sont favorisés														5		25			15		75			
Score Item 3 = 4,33/5																								
Item 4: Appréciation générale																								
19. La qualité globale de l’atelier est satisfaisante														1		5			19		95			
20. Les objectifs définis ont été atteints														2		10			18		90			
21. Vous recommandez ce séminaire à un collègue														1		5			19		95			
Score Item 4 = 4,5/5																								
Item 5: Pertinence pour la pratique																								
22. La formation m'a permis de mettre à jour mes connaissances														3		15			17		85			
23. Cette formation m’a permis d’acquérir des compétences organisationnelles pour planifier une évaluation par ECOS														2		10			18		90			
24. La séance peut améliorer ma pratique professionnelle future														3		15			17		85			
Score Item 5 = 4,5/5																								
Score moyen de satisfaction = 4,36+4,2+4,33+4,5+4,5/5 = 4,37																								
